# A dosimetric comparison of two-phase adaptive intensity-modulated radiotherapy for locally advanced nasopharyngeal cancer

**DOI:** 10.1093/jrr/rru119

**Published:** 2015-02-08

**Authors:** Imjai Chitapanarux, Kittisak Chomprasert, Wannapa Nobnaop, Somsak Wanwilairat, Ekasit Tharavichitkul, Somvilai Jakrabhandu, Wimrak Onchan, Patrinee Traisathit, Dirk Van Gestel

**Affiliations:** 1 Division of Therapeutic Radiology and Oncology, Faculty of Medicine, Chiang Mai University, 110 Intawarorose Road, Chiang Mai, 50200, Thailand; 2 Department of Statistics, Faculty of Science, Chiang Mai University, Chiang Mai, Thailand; 3 Department of Radiotherapy, University Radiotherapy Antwerp, UZA/ZNA, Antwerp, Belgium

**Keywords:** two-phase, adaptive radiotherapy, nasopharyngeal cancer, intensity-modulated radiation therapy

## Abstract

The purpose of this investigation was to evaluate the potential dosimetric benefits of a two-phase adaptive intensity-modulated radiotherapy (IMRT) protocol for patients with locally advanced nasopharyngeal cancer (NPC). A total of 17 patients with locally advanced NPC treated with IMRT had a second computed tomography (CT) scan after 17 fractions in order to apply and continue the treatment with an adapted plan after 20 fractions. To simulate the situation without adaptation, a hybrid plan was generated by applying the optimization parameters of the original treatment plan to the anatomy of the second CT scan. The dose–volume histograms (DVHs) and dose statistics of the hybrid plan and the adapted plan were compared. The mean volume of the ipsilateral and contralateral parotid gland decreased by 6.1 cm^3^ (30.5%) and 5.4 cm^3^ (24.3%), respectively. Compared with the hybrid plan, the adapted plan provided a higher dose to the target volumes with better homogeneity, and a lower dose to the organs at risk (OARs). The Dmin of all planning target volumes (PTVs) increased. The Dmax of the spinal cord and brainstem were lower in 94% of the patients (1.6–5.9 Gy, *P* < 0.001 and 2.1–9.9 Gy, *P* < 0.001, respectively). The D_mean_ of the contralateral parotid decreased in 70% of the patients (range, 0.2–4.4 Gy). We could not find a relationship between dose variability and weight loss. Our two-phase adaptive IMRT protocol improves dosimetric results in terms of target volumes and OARs in patients with locally advanced NPC.

## INTRODUCTION

Radiotherapy (RT) is the treatment of choice for nasopharyngeal cancer (NPC) because of the high radiosensitivity and surgically inaccessible anatomic location. For locally advanced NPC, several randomized controlled trials and a meta-analysis have indicated better overall survival rates with concurrent chemoradiotherapy (CCRT) compared with RT alone [1–5]. Intensity-modulated radiotherapy (IMRT) provides improved tumor target coverage with significantly being more sparing of sensitive normal tissue structures in the treatment of NPC. Randomized trials have shown a reduction in late xerostomia, resulting in an important improvement in the quality of life with IMRT compared with conventional RT [6–8]. Therefore, IMRT is considered standard of care in NPC. Recently, the randomized trial of Peng and colleagues also showed superior locoregional control with IMRT compared with conventional 2D RT [9]. In radiotherapy for NPC, vital structures (such as the salivary glands, brainstem, spinal cord, optic nerves and optic chiasm) are close to the tumor volumes. IMRT creates sharp dose gradients between the margins of the target volumes and the organs at risk (OARs). Therefore, even small changes in anatomy can result in a lower dose to (part of) the target volumes or a higher dose to the OARs. Treatment planning and quality assurance (QA) for IMRT plans requires a lot of time and adaptive strategies are still being developed. Therefore, adaptive RT indicates both additional cost to the patient and extra workload to the clinical staff. Hence, most investigators use the initial plan for IMRT for the whole course of IMRT. However, head and neck cancer (HNC) patients in general, and NPC patients in particular, were found to have changes in anatomical structures during the course of RT due to the shrinkage of the primary tumor and/or the lymph nodes, or due to changes in body contour following profound body weight loss [10–12]. Barker *et al.* [13] used repeated computerized tomography (CT) scans throughout a 7-week RT course and reported a 70% reduction in the gross tumor volumes (GTVs). They also noticed substantial changes in the anatomical structures, including external neck contour modifications, a medial shift of normal structures due to tumor shrinkage and weight loss, and also parotid shrinkage. This appeared to be significant during the course of treatment and could potentially have a dosimetric impact when highly conformal treatment techniques are used. A study by Hansen *et al.* [10] using the ‘hybrid technique’ revealed that repeated CT scans and re-planning during the course of IMRT for HNC ensured adequate doses to target volumes and better sparing of the OARs for a group of patients who experienced clinically apparent changes in anatomy. Wu *et al.* [14] suggested that the parotid glands could be better spared by re-planning using repeated CT scans during the course of RT. It may be hypothesized that the doses to the target volumes and OARs during the 6–7 week course of fractionated IMRT may significantly differ from the planning doses based on the reference CT scan images obtained before treatment. Therefore, repeat imaging and re-planning, even with a single mid-treatment scan, may improve tumor coverage and organ sparing by decreasing the impact of anatomical changes on the dose distribution during IMRT. All the studies mentioned have shown anatomic changes that could negatively affect adequate target coverage and normal organ sparing of IMRT plans. Moreover, in most studies on adaptive RT in HNC [10, 14], the primary tumor locations are heterogeneous and the patients are not balanced with respect to whether the left or right neck was treated. Although the results from these studies are largely consistent, it remains unclear whether these results are applicable to NPC, for which the anatomy is in close vicinity to a number of critical structures (brainstem, parotids, optic chiasm, optic nerves) and significantly differs from lower HNC sites. A recent study by Wang and colleagues [15] indicates their interest in this approach for NPC, but there were some shortcomings in that study. The prescribed dose to planning target volume 1 (PTV1) was only 60 Gy, and the impact of re-planning on the optic neural structures was not analyzed. Surprisingly, they did not find any impact for the important decrease in the primary target volume (30% in the GTV and 21% in PTV1) on the dose to the brainstem nearby. In this study we will evaluate the contribution of repeat CT scans and adaptive IMRT re-planning in the assessment of anatomic and volumetric changes on dosimetric outcome during the course of IMRT for loco-regionally advanced NPC patients treated with 70 Gy radiation to the PTV.

## MATERIALS AND METHODS

Between December 2011 and July 2012, patients with non-metastatic biopsy-proven Stage II–IVB (AJCC Staging 2010 [16]) loco-regionally advanced NPC, WHO types I–III, were prospectively enrolled in our study and treated with 33 fractions of IMRT. According to the Intergroup study 0099 [2], all patients were treated with concurrent cisplatin-based chemotherapy followed by three courses of adjuvant chemotherapy. Patients with a performance Status of 0–1 had WBC ≥4000/µl, a platelet count of ≥100 000/µl, and adequate renal function (as documented by serum creatinine of ≤1.5 mg/dl or calculated creatinine clearance ≥50 ml/min). The study was approved by the Ethics Committee of the hospital, and written informed consent was obtained from all patients.

### Simulation CT procedures

All patients were immobilized in a supine position with a thermoplastic head–neck and shoulder mask. A planning or reference CT scan of the head and neck region was obtained using a 3-mm slice thickness from the skull vertex to 2 cm below the clavicle. The initial simulation CT scan (CT-1) was performed within 10 days before the treatment commencement and was used to generate the original treatment plan. A second simulation CT scan (CT-2), for the adaptive plan, was acquired at the 17th fraction using the same isocenter on the original mask. If the thermoplastic mask no longer fitted tightly at the time of re-scanning, a new immobilization mask was made in the initial treatment position. Finally, both reference CT scans were transferred to a pencil beam algorithm treatment planning system (TPS) (KonRad Version 2.2.23, Siemens, Erlangen, Germany).

### Delineation of target volumes and OARs

For each patient, all target volumes and critical normal structures were manually outlined slice by slice on CT-1 and CT-2 by the same attending physician. The target volumes were delineated following the RTOG 0225 protocol [16]. The Gross Tumor Volume (GTV) was defined as all known macroscopic disease determined from CT, clinical information, and endoscopic findings. Lymph nodes were considered positive when >1 cm and/or with a necrotic center. The Clinical Target Volume (CTV) was defined as the GTV plus areas of potential microscopic disease. Three different CTVs were defined. The CTV70 contained the GTV plus a margin of 5 mm limited to air and non-involved tissues. The CTV59.4 contained the CTV70 plus a 5-mm margin plus areas at risks for microscopic involvement, including the entire nasopharynx, clivus, base of skull, parapharyngeal space, the inferior part of the sphenoid sinus and the posterior third of the nasal cavity and maxillary sinuses as well as the retropharyngeal lymph nodes, and bilateral levels IB, II, III and V lymph nodes. The CTV54 included the supraclavicular nodes. The PTV provides a margin of 5 mm around the respective CTV to compensate for the variability in treatment set-up. The OARs outlined routinely include the brainstem, spinal cord, optic nerves, optic chiasm, parotid glands, pituitary gland, mandible, eyes, temporal lobe, and glottic larynx.

For re-contouring, the adapted GTV was generated by removing the air cavity formed by tumor shrinkage, while maintaining the other dimensions of the initial primary tumor GTV [17]. The Nodal GTV was adapted if there was a clear displacement of an anatomical border (e.g. muscle or skin). The original CTVs were maintained, but adapted to the new limits of air and non-involved tissues. The OARs were re-contoured.

### Planning

The CT-1 was used to generate the original treatment plan (PLAN-1). The CT-2, performed at the 17th fraction of IMRT, was used for adaptive planning (PLAN-2). After re-planning, PLAN-2 was used from the 21st fraction onwards for the completion of the planned 33 fractions. A hybrid plan (PLAN-3) was generated for each patient by applying the beam configurations of PLAN-1 to the anatomy of the CT-2. The dose–volume histograms (DVHs) of PLAN-2 and PLAN-3 were compared in order to investigate the dosimetric advantages of adaptive planning.

### Dose prescription and optimization

A simultaneous integrated boost technique was planned and prescribed at D95 in order to deliver in 33 fractions a dose of 70 Gy (2.12 Gy/fraction) to the ‘PTV70’, a dose of 59.4 Gy (1.8 Gy/fraction) to ‘PTV59.4’ and a dose of 54 Gy (1.64 Gy/fraction) to ‘PTV54’. These target prescription doses, as well as the OARs limit doses, were adopted from the RTOG 0225 trial [16]. The total doses were divided into two portions: the first 20 fractions (delivered according to PLAN-1) and the remaining 13 fractions (delivered according to PLAN-2). The dose constraints for each portion of the treatment are shown in Table 1. The IMRT plans were generated and approved for each patient using KonRad Version 2.2.23 (Siemens, Erlangen, Germany) with seven 6-MV photon beams. All plans were designed as step-and-shoot IMRT for delivery on a Primus linear accelerator (Siemens, Erlangen, Germany) with 10-mm leaf thickness. The original treatment plan (PLAN-1) was generated on the initial simulation CT scan (CT-1) and delivered for the first 20 fractions. The second simulation CT scan (CT-2) was used to generate the adapted plan (PLAN-2) that was used in the remaining 13 fractions to complete the planned course of IMRT. By applying the fluence map of the original plan (PLAN-1) to the anatomy of CT-2 without re-optimization, a hybrid IMRT plan (PLAN-3) was generated for each patient to represent the dose distribution if no re-planning had been done.

**Table 1.** The dose constraints for the target volumes and organs at risk (OARs)

Target volumes and OARs	PLAN-1 (Gy)	PLAN-2 (Gy)	Total (Gy)
PTV_70_	42.40	27.56	69.96
PTV_59.4_	36.00	23.40	59.40
PTV_54_	32.80	21.32	54.12
Brainstem D_5_	32.70	21.30	54.00
Optic nerves D_5_	32.70	21.30	54.00
Optic chiasm D_5_	32.70	21.30	54.00
Spinal cord D_5_	27.30	17.70	45.00
Mandible D5	42.40	27.60	70.00
Temporal lobes D_5_	36.40	23.60	60.00
Eyes D_mean_	21.20	13.80	35.00
Glottic larynx D_mean_	27.30	17.70	45.00
Parotid gland, at least one			
- D_mean_	15.80	10.20	26.00
- D_50_	18.20	11.80	30.00

PLAN-1 = initial plan, PLAN-2 = adapted plan on reference scan after 17 days of treatment, Gy = Gray, PTV*_X_* = planning target volume prescribed to *x* Gy, D = dose, D*_x_* = dose to *x*% of the volume.

### Analysis

The volume of targets and OARs was compared between CT-1 and CT-2 using paired sample analysis, and the DVHs were calculated. The hybrid technique reported by Hansen *et al.* [10] was used to evaluate the dosimetric difference between PLAN-2 and PLAN-3. This comparison accounted only for the last 13 fractions. To assess the target dose homogeneity of each plan, the homogeneity index (HI) has been calculated. The HI of the PTV is defined as the ratio of D5 to D95; D5 and D95 correspond to the dose delivered to 5% and 95% of the PTV, respectively [9, 18–19]. A HI value approaching 1 is generally regarded as a favorable target homogeneity. The weight of all patients was recorded weekly, and the mean weight loss was calculated. The correlation between the percentage of weight loss and volumetric/dosimetric changes in target volumes and OARs was analyzed.

### Statistical analysis

The Statistical Package for the Social Science (SPSS Statistics 20.0, SPSS Inc., Chicago, IL, USA) was used for statistical analysis. The Kolmogorov–Smirnov test was used to test data for normality. Comparisons between the two plans were performed by analysis of a paired sample *t*-test if the distribution of the difference between the groups was normally distributed. Otherwise, the non-parametric Wilcoxon signed-rank test was used. A *P*-value < 0.05 indicated the difference as statistically significant. Pearson's correlation coefficient was used to analyze the correlation between weight loss and volumetric/dosimetric changes in target volumes and OARs when the sampling distribution was normally distributed.

## RESULTS

### Patient characteristics

Between December 2011 and July 2012, a total of 17 patients (median age, 46 years; range, 18–62 years) with non-metastasized Stage II–IVB NPC treated with IMRT were prospectively enrolled in this study (AJCC Staging, 2010, seventh edition [20]). Of these, 13 were male and 4 were female, and all had non-keratinizing carcinoma of undifferentiated subtype. Four patients had Stage II disease, seven Stage III, four Stage IVA, and two Stage IVB. In the form of CCRT, five patients received three cycles of cisplatin as planned, but 12 received only two cycles because of acute toxicity and patient refusal. Mean weight loss during the treatment course was 8.4 kg (range, 3–15 kg).

### Volume comparisons

Differences in target and OARs volume between the initial and second reference CT scans are listed in Table 2. All adapted PTVs showed a significant volume diminution compared with the initial PTVs. The average volume reduction of the parotid glands was 30.5% and 24.3% for the ipsilateral and contralateral parotid gland, respectively. Between the consecutive CT scans, there were no significant differences observed in the volume of other contoured normal structures, including the spinal cord, brainstem, optic nerves, optic chiasm and eyeballs.

**Table 2.** The mean volume of targets and organs at risk (OARs) on CT-1 and CT-2

Target and OARs	CT-1(cm^3^)	CT-2 (cm^3^)	% change	*P*-value
GTV	77.6	63.3	−18.4	0.001
CTV_70_	164.1	152.0	−7.4	0.001
CTV_59.4_	468.3	450.9	−3.7	0.004
CTV_54_	125.5	107.6	−14.3	0.003
Ipsilateral parotid gland	20.0	13.9	−30.5	<0.001
Contralateral parotid gland	22.2	16.8	−24.3	<0.001

CT-1 = initial reference scan, CT-2 = reference scan after 17 days of treatment, GTV = gross tumor volume, CTV*_X_* = clinical target volume prescribed to *x* Gy.

### Dosimetric comparisons

**Fig. 1.**
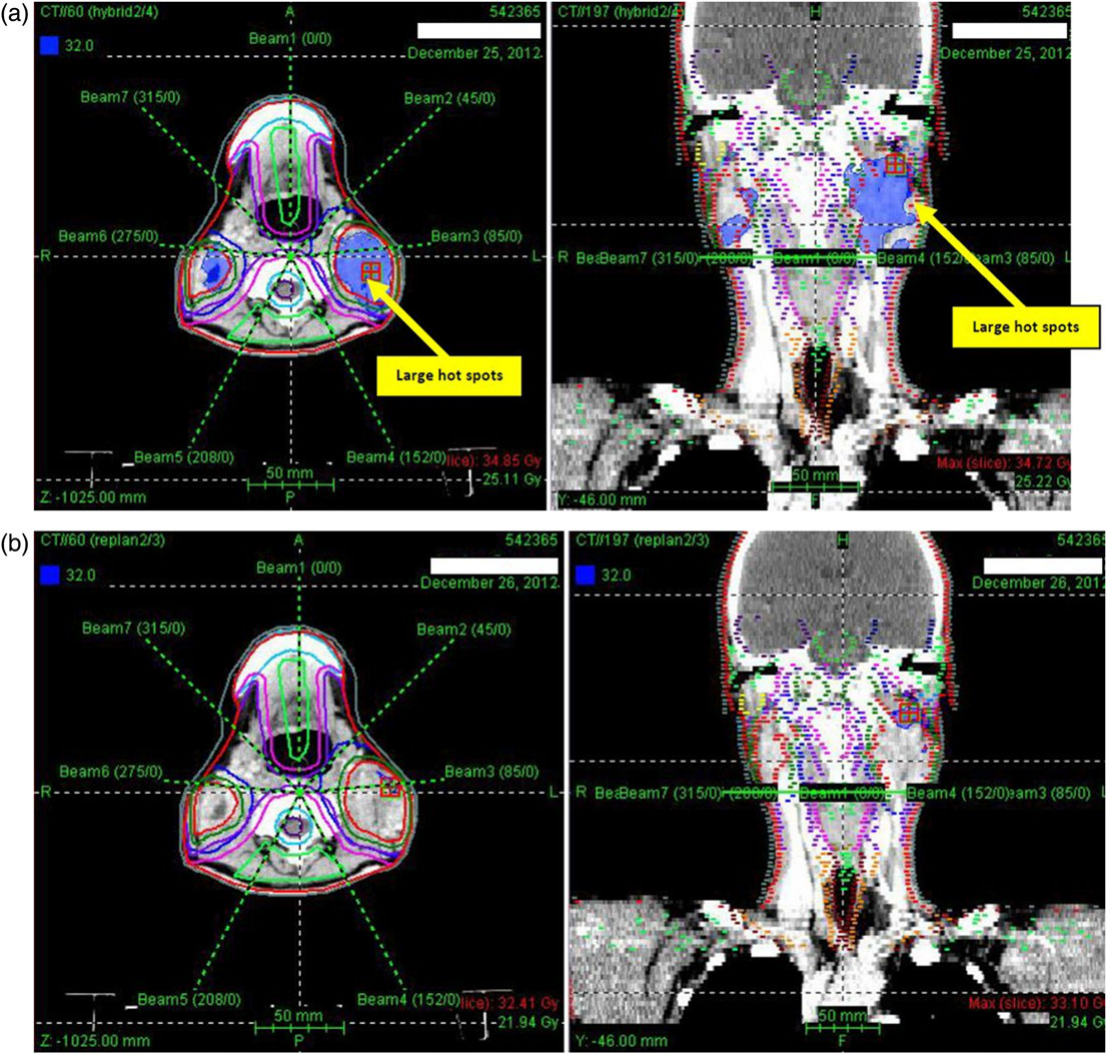
Example of a 37-year-old man with T_1_N_2_M_0_ nasopharyngeal cancer. Large areas of ≥115% of the prescribed dose (blue areas) within the high-risk planning target volume (PTV_70_) are observed in PLAN-3 (without re-planning) (**a**). In PLAN-2 (after re-planning), the hot spots in the target volume are decreased (**b**).

**Fig. 2.**
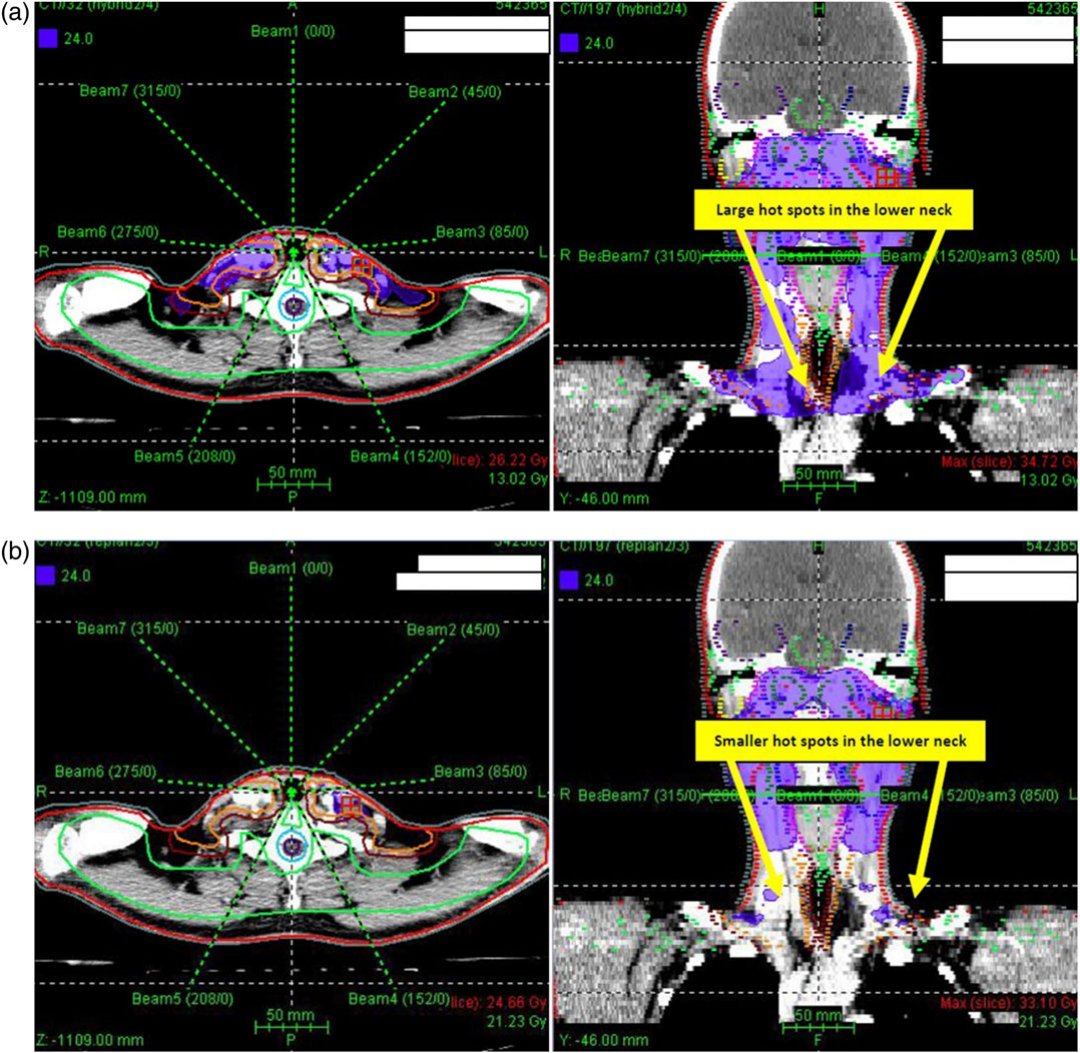
In the second portion of treatment of the same patient as in Fig. 1, the hybrid plan shows large areas of ≥115% of the prescribed dose (violet areas at the lower neck) within the low-risk planning target volume (PTV_54_) without re-planning (**a**). After re-planning, the hot spots within the PTV_54_ are smaller (**b**).

**Fig. 3.**
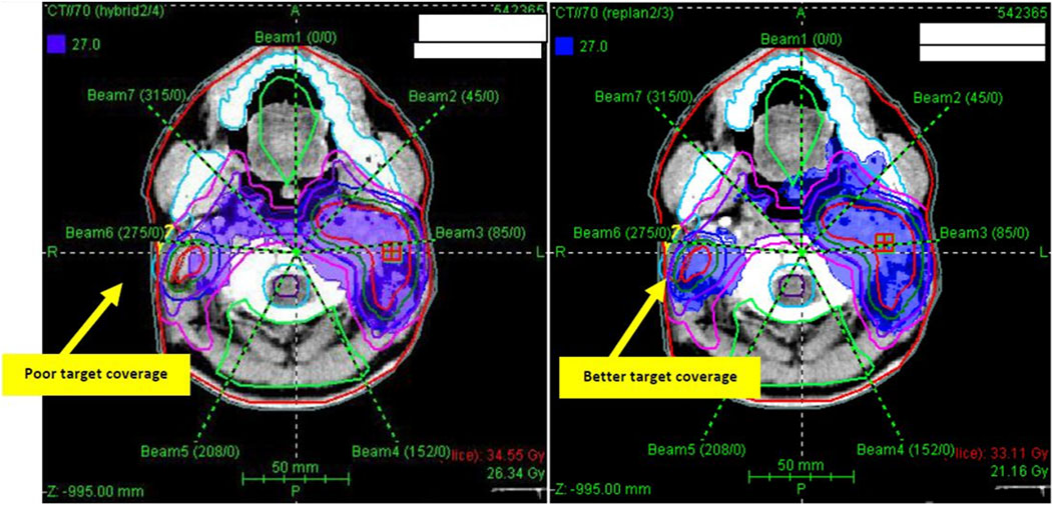
The left image, shows the isodose areas (dark blue surface) obtained without re-planning. Even the gross tumor volume (red contour) in the right neck is not properly covered by the isodose surface. The right image shows the same slice with the isodose areas obtained by re-planning. The area of underdose within the PTV_70_ (dark blue contour) without re-planning is better and more conformally covered with re-planning.

For the adapted PLAN-2, the mean dose for all targets was higher and the mean volume of hot (V115) and cold (V93) spots decreased and demonstrated a better dose homogeneity (lower mean HI) compared with the hybrid PLAN-3 (Table 3 and Figs 1–3). Whereas the target volume doses increased in the adapted plan, the doses to some critical OARs, such as the spinal cord, the brainstem and the contralateral parotid decreased (Table 3). For the spinal cord, re-planning decreased the mean Dmax and D5 by 4.6 Gy and 3.8 Gy, respectively (*P* < 0.001), and 70% of the patients exceeding the Dmax tolerance dose of 45 Gy in PLAN-3 met this constraint in PLAN-2. Furthermore, for the brainstem, the mean Dmax and D5 were significantly higher in PLAN-3 than in PLAN-2, by 3.4 Gy and 2.8 Gy, respectively (*P* < 0.001). Without re-planning, the Dmax of the spinal cord and of the brainstem increased in 94% of patients (*P* < 0.001), by 2.1–9.9 Gy and 1.6–5.9 Gy, respectively. For the contralateral parotid gland, all dosimetric endpoints decreased in the adapted PLAN-2, including the mean Dmean and D50 (*P* = 0.045 and 0.021, respectively). Moreover, 13% of the patients presenting with a D50 of the contralateral parotid gland exceeding the respective tolerance dose of 30 Gy in PLAN-3 could be ‘saved’ by PLAN-2. Although the mean Dmean of the contralateral parotid gland could be significantly reduced with re-planning, the dose constraint of 26 Gy could not be achieved in all patients. The differences between both plans in the mean dosimetric endpoints for the ipsilateral parotid gland, the eyeballs, optic nerves and optic chiasm were not statistically significant.

**Table 3.** Dosimetric comparison between the original PLAN-1, the adapted PLAN-2 and the hybrid PLAN-3

Parameters	PLAN-1 (Mean ± SD)	PLAN-2 (Mean ± SD)	PLAN-3 (Mean ± SD)	*P*-value of PLAN-2 and PLAN-3
D_95_ (Gy)				
GTV	44.8 ± 0.6	74.9 ± 2.0	73.6 ± 0.9	0.046
CTV_70_	44.0 ± 0.7	72.7 ± 1.0	72.0 ± 1.2	<0.001
PTV_70_	41.9 ± 1.0	69.0 ± 1.1	68.4 ± 1.7	0.013
CTV_59.4_	37.7 ± 0.4	62.3 ± 0.7	61.2 ± 0.9	<0.001
PTV_59.4_	35.7 ± 0.4	58.8 ± 0.6	57.1 ± 0.7	<0.001
CTV_54_	33.3 ± 0.5	55.1 ± 0.6	53.9 ± 1.6	0.029
PTV_54_	32.5 ± 0.4	53.7 ± 0.5	52.1 ± 1.8	0.004
V_115_ (%)				
PTV_70_	2.9 ± 1.6	3.3 ± 1.7	21.2 ± 9.5	<0.001
PTV_54_	1.3 ± 1.2	2.0 ± 11.7	16.9 ± 12.3	<0.001
V_93_ (%)				
PTV_70_	0.9 ± 0.5	1.5 ± 0.9	2.9 ± 1.5	0.016
PTV_59.4_	1.6 ± 0.7	1.7 ± 0.7	5.4 ± 1.5	<0.001
PTV_54_	0.4 ± 0.7	0.6 ± 1.1	5.6 ± 4.7	<0.001
Homogeneity Index				
PTV_70_	1.16 ± 0.03	1.16 ± 0.02	1.19 ± 0.03	<0.001
PTV_59.4_	1.34 ± 0.02	1.34 ± 0.02	1.40 ± 0.02	<0.001
PTV_54_	1.14 ± 0.05	1.15 ± 0.06	1.20 ± 0.06	<0.001
Spinal cord D_max_	27.2 ± 1.3	44.5 ± 1.8	49.1 ± 1.9	<0.001
D_5_	24.2 ± 1.7	39.7 ± 2.4	43.5 ± 1.7	<0.001
Brainstem D_max_	37.6 ± 3.3	61.8 ± 4.9	65.2 ± 4.8	<0.001
D_5_	33.7 ± 3.2	55.5 ± 4.7	58.3 ± 5.1	<0.001
Contralateral parotid gland D_mean_	19.7 ± 2.1	32.7 ± 3.3	33.8 ± 3.1	0.045
D_50_	17.2 ± 1.7	28.9 ± 3.0	30.1 ± 2.5	0.021
Ipsilateral parotid gland D_mean_	24.6 ± 5.1	40.9 ± 8.0	40.0 ± 8.1	0.298
D_50_	22.6 ± 5.8	37.3 ± 9.1	38.4 ± 9.4	0.328
Ipsilateral optic nerve D_max_	30.7 ± 10.6	46.4 ± 17.5	46.6 ± 19.7	0.845
D_5_	29.2 ± 10.6	44.3 ± 17.2	45.0 ± 19.7	0.631
Contralateral optic nerve D_max_	25.2 ± 9.0	44.8 ± 11.2	45.0 ± 13.1	0.905
D_5_	24.1 ± 9.1	41.6 ± 11.8	42.7 ± 13.9	0.441
Optic chiasm D_max_	31.4 ± 8.2	51.5 ± 11.5	53.0 ± 12.3	0.097
D_5_	30.8 ± 8.4	50.2 ± 11.7	51.4 ± 12.6	0.214

PLAN-1; 2.12 Gy in 20 fractions to PTV70, PLAN-2 and PLAN-3; 2.12 Gy in PLAN-1+13 fractions to PTV70. SD = standard deviation, GTV = gross tumor volume, CTV*_X_* = clinical target volume prescribed to *x* Gy, PTV*_X_* = planning target volume prescribed to *x* Gy, V_x_ = volume receiving *x*% of the prescribed dose, D*_x_* = dose to *x*% of the volume.

### Effects of weight loss

All patients experienced weight loss, with a mean loss of 9.8% (2.1–25.0%) of body weight. This weight loss was only significantly correlated with the change in the D95 of the PTV70 (Pearson's correlation coefficient = 0.544, *P* = 0.024) in PLAN-3 (Table 4). However, the percentage of weight change did not significantly correlate with any of the other changes in volume or dose.

**Table 4.** Correlations between weight loss and volume shrinkage or dosimetric variability of the targets and organs at risk

	Correlation coefficient	*P*-value
**Weight loss and volume shrinkage:**		
GTV	0.247	0.340
Ipsilateral parotid	0.057	0.834
Contralateral parotid	0.012	0.964
**Weight loss and dosimetric changes:**		
PTV_70_	0.544	0.024
PTV_59.4_	0.480	0.051
PTV_54_	−0.255	0.340
Spinal cord (D_max_)	0.295	0.250
Brainstem (D_max_)	0.131	0.616
Ipsilateral parotid (D_mean_)	−0.349	0.185
Contralateral parotid (D_mean_)	0.244	0.344

Pearson's correlation coefficient was used. GTV = gross tumor volume, PTV*_X_* = planning target volume prescribed to *x* Gy, D = dose, max = maximum.

## DISCUSSION

Patients receiving RT to the head and neck region are likely to experience significant anatomical changes during the 6 to 7 week treatment course as a result of tumor and/or nodal shrinkage and due to contour changes following weight loss. When significant anatomic changes occur, not only the dose distribution to both targets and OARs will no longer be valid, but also the patient immobilization mask will no longer match the current facial contour, leading to an increase in dose deviations. In our study, the mean volume of all targets and of the bilateral parotid glands was significantly smaller after 17 fractions (at the time of CT-2), and corresponding dosimetric changes were found. This corresponds with the findings of Barker *et al.* [13], who conducted a pilot study to quantify the magnitude of these anatomic changes using systematic CT imaging. They enrolled 14 patients with head and neck cancer and performed CT scans three times a week during the course of RT. The GTV decreased throughout the course of radiotherapy at a median rate of 1.8% per day; the parotid glands also decreased in volume at a median rate of 0.6% per day and shifted medially (median, 3.1 mm; range, 0–9.9 mm) with time. All of these suggested that there were considerable volume changes, resulting in dosimetric changes in later fractions of the treatment courses. Therefore, they indicated that if anatomic changes cause suboptimal target coverage or increase the dose to normal tissue over time, an adaptive radiotherapy strategy should be designed to maintain good target coverage while protecting the sensitive normal tissues from high radiation doses.

Local control is directly related to the dose delivered to the target volumes for NPC. Cold and hot spots induced by anatomic and volumetric changes may result in a significant dose inhomogeneity. Limited published data are available on target volume dose variations for NPC patients undergoing IMRT. The results of the current study are consistent with others conducted on non-NPC head and neck cancer patients. Hansen *et al.* [10] presented an analysis of 13 locally advanced HNC patients receiving IMRT, in which all patients underwent a repeat CT scan and re-planning with an average interval of 39 days (average of 19 treatment fractions delivered) between the first treatment fraction and the second CT imaging. They found that the D95 of the GTV and the CTV was reduced in 92% of patients, by 0.8–6.3 Gy (*P* = 0.02) and by 0.2–7.4 Gy (*P* = 0.003), respectively for the hybrid plan. We found a comparable dose increase to the D95 of the PTV70, PTV59.4 and PTV54 of 0.2–2.6 Gy, 0.4–2.7 Gy and 0.2–7.0 Gy, respectively, in our adapted plan. Therefore, we concluded that the adaptive plan is beneficial in terms of ensuring the prescribed doses to target volumes and for improving target dose homogeneity. However, the results above are in contrast to a recent study of Wang *et al.* [15] in NPC patients, as they did not find any significant difference in target coverage between the different plans. This can be explained by their rather large reductions in target volumes for CT-2 (30–45% for the GTV and 21% for the CTV versus 18% and 7%, respectively, in our study) as one can expect to find the dose reductions mainly in the periphery of the target volumes.

The average volume reduction of the parotid glands found in this study was 30.5% and 24.3% for the ipsilateral and contralateral parotid gland, respectively. This is in agreement with Baker *et al.* [13] (who found a parotid shrinkage of 28%) and is slightly more than found by X Wang *et al.* [11] and R Wang *et al.* [15]. The dosimetric changes observed in our study were also largely consistent with the results of other studies. Some studies reported that the lateral border of both parotid glands contracted medially, which resulted in increasing the mean parotid dose [11, 21]. Robar *et al.* found that the Dmean of the left and right parotids increased by 2.6% and 0.3%, respectively [21], whereas the results from X Wang *et al.* [11] showed an increase of 7.0% and 8.3%, respectively [12]. R Wang and colleagues even reported an increase in the mean parotid dose of ∼13% [15]. The present study found an increase in the Dmean of the ipsilateral and contralateral parotids of 2.1% and 3.3%, respectively. However, our results, as well as those of R Wang *et al*., have shown that this increased dose to the parotids can be (partially) counterbalanced by an adaptive plan after 20 sessions, decreasing significantly the mean Dmean of the contralateral parotid gland for the remaining sessions. This correction did not occur in the ipsilateral parotid gland because this parotid gland is often located adjacent to or even overlapping with the high-risk PTV, which should not be adapted. The mean Dmax of the spinal cord and brainstem were lower in the adaptive plan than in the hybrid plan in 94% of patients, which is consistent with previous reports [10–12, 23]. Because the brainstem is immediately adjacent to the GTV/PTV of the primary tumor in NPC, a slight change in anatomy due to weight loss or tumor shrinkage may increase the brainstem dose above the constraint criteria. Consequences of an overdose to the brainstem might be huge, stressing the possible role of adaptive RT in high-dose IMRT to this region. Surprisingly, a recent study in NPC by R Wang *et al*. did not find a higher dose to the brainstem in the hybrid plan, only a higher dose to the spinal cord [15]. In contrary to that of the brainstem and the spinal cord, the maximum dose to the optic nerves and the optic chiasm (also critical OARs in the direct vicinity of the nasopharynx) did not significantly change between plans 2 and 3. This might be explained by the position of these structures at the cranial edges of the target volume, separated from the target volume by bony structures that change very little during tumor regression.

Weight loss could not reliably predict the dosimetric changes for most PTVs, spinal cord, brainstem or parotid glands. This might be due to other confounding effects, such as positional variability, even when attempts were made to restore patient positioning to the same as it had been in the initial CT scan. However, fast weight loss is often due to catabolism and loss of muscles. Hence, this might influence the position of the thorax, neck and shoulders but to a much lesser extent the position/dose of a primary tumor at the base of skull. Moreover, for NPC patients with large metastatic cervical lymph nodes, a dramatic regression of the lymph nodes during treatment could affect the dose distribution, irrespective of the weight loss. Although the doses to tumor volumes would have increased and the doses to OARs would have decreased with an adaptive plan, it remains unclear whether these dosimetric changes would translate into favorable clinical outcomes. The clinical outcome for these patients was not presented in this report because of the short follow-up time. Larger, randomized studies are warranted to evaluate the clinical impact of dosimetric changes by an adaptive plan. Another important concern is how to delineate target volumes on repeat CT scans obtained during the course of IMRT. Since cancer is heterogeneous, tumor volumes are not reflective of the amount of actively replicating cancerous tissue [25]. It is not clear whether shrinking tumors leave behind nests of cells that should be treated further, or whether smaller fields adequately encompass subclinical disease. Furthermore, re-contouring of large lymph nodes that regress during therapy has the possible risk of missing extracapsular spread below the detection threshold. Sparing of these high-risk zones might be detrimental for tumor control [16, 24, 25]. Hansen *et al.* [10] choose to maintain the size of the original GTV when contouring the GTV on the anatomy of the second CT scans. In our study, for the reasons mentioned above, we respected the original CTVs as much as possible. Nevertheless, removing the air cavity formed by tumor shrinkage [16] and adaptation of lymph node volumes to changed anatomical borders (e.g. muscles and skin) resulted in a mean decrease of 18% in the GTV volume, while simple adaptation of the original CTVs to changes in anatomic structures resulted in a mean volume decrease of 7% in CTV70, of 4% in CTV59.4 and of 14% in CTV54.

We re-planned at the fourth week for three reasons. First, Barker *et al.* [13] and Bhide *et al.* [26] noticed that reduction in parotid volumes reaches a plateau at Weeks 3–4 during IMRT for HNC. Second, repeated CT scan and re-planning is laborious in our busy radiation center, increasing the workload for physicists, dosimetrists and radiation oncologists. And last but not least, Wu *et al.* [14] found that there was no need for re-planning more than once a week and that the improvement in parotid sparing saturated quickly beyond two re-plannings at the second and the fourth weeks. The most important questions remaining to be answered are the optimal timing of the second scan for the re-planning and whether a third re-planning would be necessary in the treatment of loco-regionally advanced NPC. To date, no prospective clinical study has been performed to demonstrate the clinical benefits and practicality of this approach. There are several aspects of this study that require further investigation. Due to the small number of patients in this study, we were unable to separate the effects of tumor shrinkage from those of weight loss on dosimetric outcome. Future study with more frequent and convenient imaging (such as with an integrated in-room CT-linear accelerator or with kV or MV conebeam CT) will be necessary to further investigate the relationships between patient positioning uncertainty, anatomical changes, and dosimetric changes to target volumes and normal tissues. With more frequent imaging, it will be technically challenging to obtain the cumulative DVHs from multiple plans based on different image sets. Further studies are needed to identify specific predictive factors that may indicate the need for re-imaging and re-planning in cases where dosimetric changes are likely to translate into changes in clinical outcome. The clinical benefit of adaptive radiation therapy in IMRT for NPC, as well as the establishment of a predictive model (cut-off value) for optimal timing of re-planning, should be investigated in an extensive prospective study. Ultimately, better and faster software is warranted to optimize the different steps of the adaptive process, making adaptive radiotherapy accessible for a broader range of patients, with daily on-line adaptive radiotherapy being the ultimate goal.

## CONCLUSION

The two-phase adaptive radiotherapy protocol in NPC shows improved dosimetric results to the target volumes and OARs. Therefore, this protocol is recommended for the treatment of NPC patients, as most of them will suffer from significant weight loss and tumor shrinkage. Future prospective studies with larger sample sizes are needed to determine criteria for the appropriate timing of repeat CT scanning and re-planning and for the selection of NPC patients that will benefit from this approach.

## FUNDING

Funding by Faculty of Medicine, Chiang Mai University. Funding to pay the Open Access publication charges for this article was provided by Faculty of Medicine, Chiang Mai University.
